# Role of the Pbrm1 subunit and the PBAF complex in Schwann cell development

**DOI:** 10.1038/s41598-022-06588-8

**Published:** 2022-02-16

**Authors:** Vanessa Polanetzki, Franziska Fröb, Tina Baroti, Margit Schimmel, Ernst R. Tamm, Michael Wegner

**Affiliations:** 1 Institut für Biochemie, Friedrich-Alexander-Universität Erlangen-Nürnberg, Fahrstrasse 17, 91054 Erlangen, Germany; 2 Institut für Humananatomie und Embryologie, Universität Regensburg, Regensburg, Germany

**Keywords:** Gliogenesis, Myelin biology and repair, Glial biology, Schwann cell

## Abstract

Myelin sheath formation in the peripheral nervous system and the ensuing saltatory conduction rely on differentiated Schwann cells. We have previously shown that transition of Schwann cells from an immature into a differentiated state requires Brg1 that serves as the central energy generating subunit in two related SWI/SNF-type chromatin remodelers, the BAF and the PBAF complex. Here we used conditional deletion of Pbrm1 to selectively interfere with the PBAF complex in Schwann cells. Despite efficient loss of Pbrm1 early during lineage progression, we failed to detect any substantial alterations in the number, proliferation or survival of immature Schwann cells as well as in their rate and timing of terminal differentiation. As a consequence, postnatal myelin formation in peripheral nerves appeared normal. There were no inflammatory alterations in the nerve or other signs of a peripheral neuropathy. We conclude from our study that Pbrm1 and very likely the PBAF complex are dispensable for proper Schwann cell development and that Schwann cell defects previously observed upon Brg1 deletion are mostly attributable to altered or absent function of the BAF complex.

## Introduction

Schwann cells represent the main population of glial cells in the peripheral nervous system (PNS) and line axons in the peripheral nerves as myelinating or non-myelinating Schwann cells. Their development from the neural crest is coordinated by combinatorially acting, continuously present or stage-specific transcription factors that form an evolving gene regulatory network in cooperation with regulatory RNAs and various classes of chromatin-modifying proteins or complexes, such as histone modifying enzymes, DNA methyltransferases and chromatin remodeling complexes^1–4^.

Among chromatin remodeling complexes, several have been studied in Schwann cells. This includes the Chd4-containing NuRD complex, the Ep400/Tip60 complex and Brg1-containing complexes of the SWI/SNF-type^5–7^. The in vivo impact of chromatin remodelers in higher organisms is usually studied by deleting a central component of the complex, frequently the ATP-hydrolyzing, energy generating subunit. In case of the SWI/SNF-type remodelers, we previously deleted the ATP-hydrolyzing Brg1 subunit in the late Schwann cell precursor stage and observed a radial sorting defect. Schwann cells did not activate the late stage transcription factors Oct6 and Egr2 and failed to transit from the immature into the pro-myelinating and further on into the myelinating Schwann cell stages^7^. Our findings thus established an essential role of SWI/SNF-type chromatin remodelers in late stage Schwann cell development.

SWI/SNF-type remodelers are intrinsically heterogeneous in their subunit composition and are frequently grouped into BAF and PBAF complexes in vertebrates^8^. BAF complexes contain either Brg1 or its close relative Brm as the ATP-hydrolyzing subunit, PBAF complexes exclusively Brg1. Better than by their ATP-hydrolyzing component, both complexes can be distinguished by signature subunits that occur only in one of the complexes. As an example, Arid1a/b subunits are specific for BAF complexes. Pbrm1 (also known as Polybromo-1, BAF180 and PB1) and Arid2 are regarded as signature subunits of PBAF complexes. Pbrm1 contains six consecutive bromodomains that serve as readers for various acetylated histones, followed by a DNA-binding high-mobility-group domain^9^. Pbrm1 is thus likely a major determinant of the DNA-binding specificity and thus of fundamental properties of PBAF complexes. Its essential role is documented by frequent loss-of-function mutations in several tumors, most prominently in clear renal cell carcinomas, its function as a tumor suppressor, its essential role in the potentiation of transcriptional activation by nuclear receptors as well as severe placental and cardiac defects upon Pbrm1 loss in the embryo^10–12^.

Considering that all essential and constitutive subunits of BAF and PBAF complexes occur in developing Schwann cells^1,13,14^ it is currently not clear, to what extent the previously observed defects in Schwann cell development after Brg1 deletion are due to impaired function of the Brg1-containing BAF complex as opposed to the equally Brg1-containing PBAF complex.

To shed light onto the role of the PBAF complex, we here chose to delete the essential PBAF subunit Pbrm1 from Schwann cells and study their development. From the absence of major alterations in lineage progression, precursor cell survival and expansion as well as terminal differentiation and myelination, we conclude that Pbrm1 is of minor relevance in developing Schwann cells. In comparison to PBAF complexes, the Brg1-containing BAF complexes therefore appear to be the major SWI/SNF-type chromatin remodelers in Schwann cells.

## Results

### Pbrm1 expression in Schwann cells

**Figure 1:**
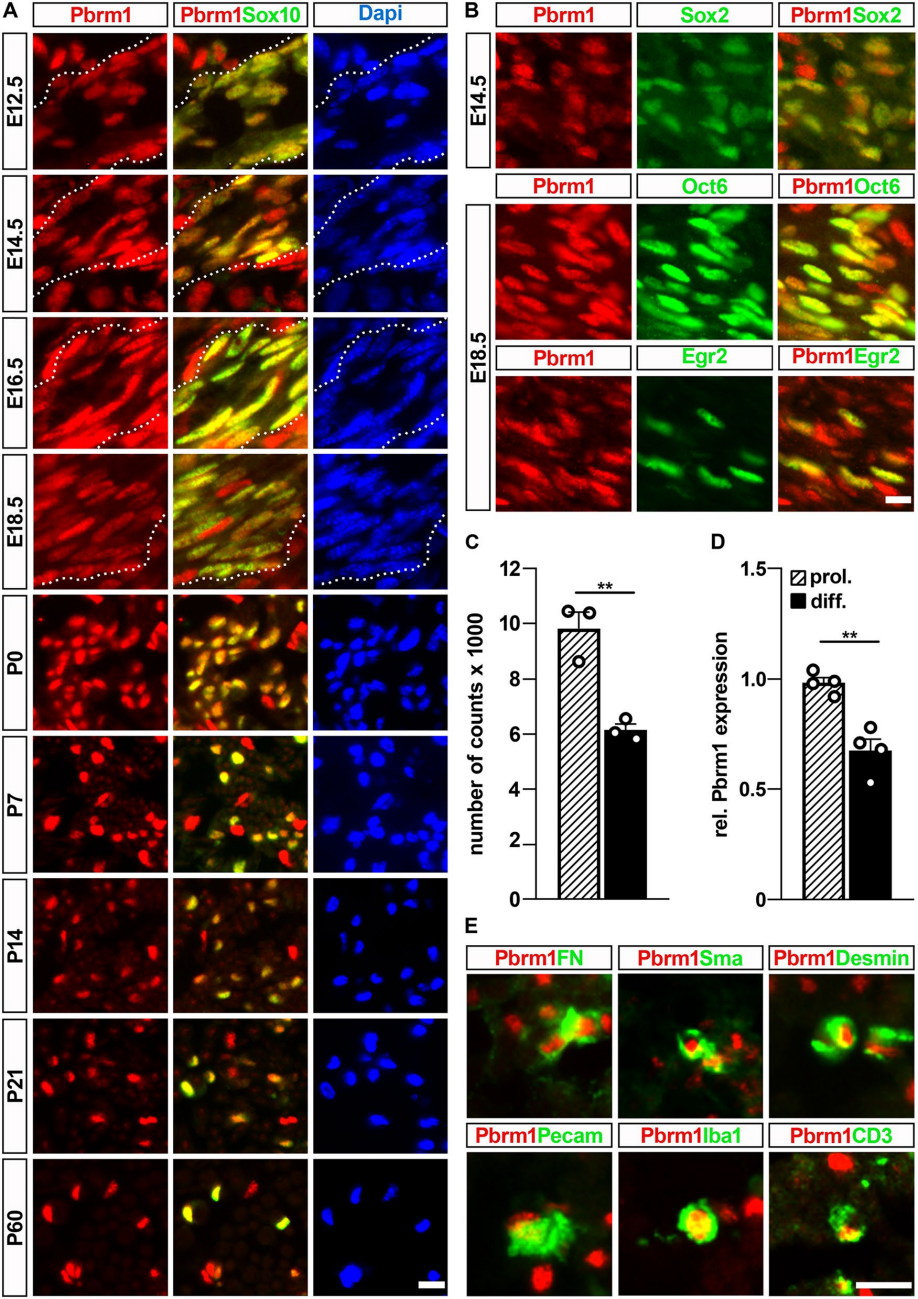
Pbrm1 occurrence in peripheral nerves and Schwann cells. (**A**) Co-immunohistochemistry with antibodies directed against Pbrm1 (red, left and middle panels) and Sox10 (green, middle panels) in embryonic spinal nerves (boundaries depicted by dotted lines) at E12.5, E14.5, E16.5 and E18.5 as well as postnatal sciatic nerves at P0, P7, P14, P21 and P60. Nuclei were counterstained with DAPI (blue, right panels). (**B**) Co-immunohistochemistry with antibodies directed against Pbrm1 (red, left and right panels) and Sox2, Oct6 and Egr2 as stage-specific Schwann cell markers (green, middle and right panels) at E14.5 and E18.5 as indicated. (**C**, **D**) Analysis of *Pbrm1* expression in Schwann cells cultured under proliferating (hatched bars) and differentiation (black bars) conditions according to published RNA-Seq data (GSE101153, presented as absolute number of counts for n = 3, in C) and quantitative RT-PCR (with average normalized expression levels in proliferating cells set to 1 and shown as mean ± SEM for n = 4, in D). Statistical significance was determined by unpaired two-tailed Student’s *t*-test (**P ≤ 0.01). (**E**) Co-immunohistochemistry with antibodies directed against Pbrm1 (red) and various markers for other nerve-associated cell types (green), including fibronectin (FN), α-smooth muscle actin (Sma), Desmin, Pecam, Iba and CD3 in sciatic nerve tissue at P14 Scale bars: 10 µm (**A**, **B**, **E**).

To be able to study Pbrm1 on the protein level, we generated an antibody against amino acids 1288–1392 of mouse Pbrm1. By immunohistochemistry, Pbrm1 was detected along embryonic spinal nerves from E12.5 until E18.5 and in sciatic nerves from birth into adulthood in all cells that were positive for the pan-Schwann cell marker Sox10 (Fig. 1A). Using co-immunohistochemistry with stage-specific markers, we were further able to show that Pbrm1 occurs in Sox2-positive immature Schwann cells as well as in Oct6-positive pro-myelinating and Egr2-positive myelinating Schwann cells (Fig. 1B). Published RNA-Seq data^13^ (Fig. 1C) and our own quantitative RT-PCR experiments (Fig. 1D) furthermore confirmed that *Pbrm1* is expressed in Schwann cells cultivated under proliferating and differentiating conditions with levels decreasing approximately by one third during differentiation.

Within the peripheral nerve, Pbrm1 was not restricted to Schwann cells, but equally occurred in other cell types. This included fibronectin-positive fibroblasts, α-smooth muscle actin-positive perivascular smooth muscle cells, Desmin-positive pericytes, Pecam-positive endothelial cells, Iba1-positive macrophages and CD3-positive T lymphocytes (Fig. 1E).

### Phenotypic consequences of Schwann cell-specific Pbrm1 deletion

To study the role of Pbrm1 in Schwann cell development, we combined a *Pbrm1* floxed allele^15^ in the homozygous state with a *Dhh::Cre* that has been previously shown to efficiently delete in the Schwann cell lineage starting at the late precursor cell stage^16^. The resulting mice are referred to as Pbrm1 cKO mice.

**Figure 2:**
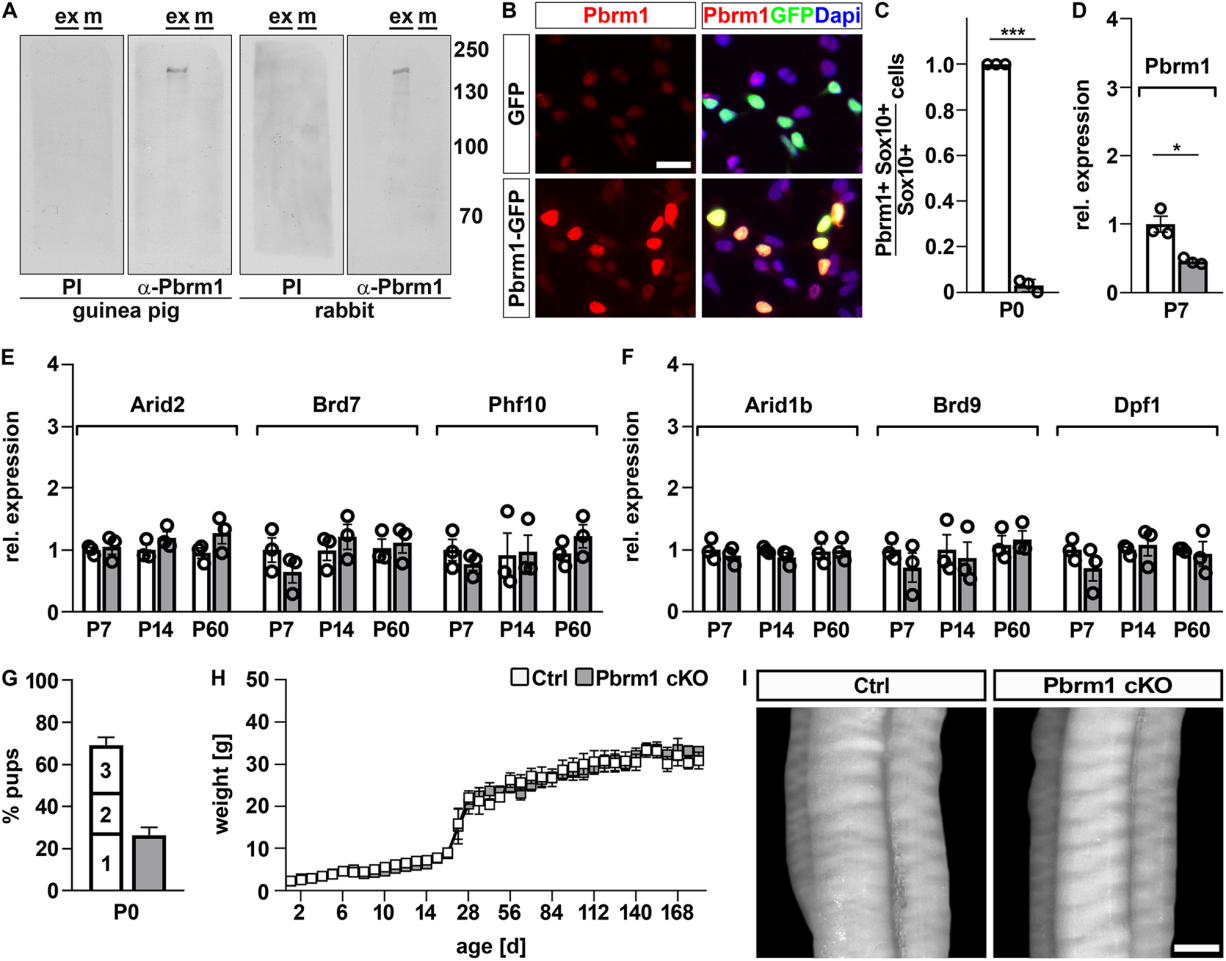
General characterization of Pbrm1 cKO mice. (**A**) Detection of Pbrm1 in Pbrm1-transfected HEK293 whole cell extracts (ex) after gel electrophoresis alongside a protein size standard (m) by western blot using guinea pig and rabbit Pbrm1 antisera (α-Pbrm1) as well as matched preimmune sera (PI) as indicated below the blots. Numbers on the right represent molecular weight markers in kilodaltons. The complete nitrocellulose membranes that were incubated with the antibodies are shown. (**B**) Immunocytochemical staining of HEK293 cells transfected with GFP (green) or a Pbrm1-GFP fusion protein (red). Nuclei are counterstained with DAPI. (**C**) Efficiency of Pbrm1 deletion as determined by the fraction of Pbrm1-positive Schwann cells among all Schwann cells in spinal nerves of control (Ctrl) and Pbrm1 cKO mice at P0 (shown as mean ± SEM, n = 3 per genotype). (**D**–**F**) Determination of transcript levels for wildtype *Pbrm1* (**D**), the PBAF-specific subunits *Arid2, Brd7, Phf10* (**E**) and the BAF-specific subunits *Arid1b, Brd9, Dpf1* (**F**) in spinal nerves of control and Pbrm1 cKO mice at P7, P14 and P60 (mean ± SEM, n = 3 per genotype). (**G**) Frequency of Pbrm1 cKO mice in litters (n = 16) obtained from breedings of *Pbrm1*^*fl/fl*^ with *Pbrm*^+*/fl*^* Dhh::Cre* mice as compared to controls (1 = *Pbrm*^+*/fl*^, 2 = *Pbrm*^*fl/fl*^*,* 3 = *Pbrm*^+*/fl*^* Dhh::Cre*). (**H**) Determination of body weight of control and Pbrm1 cKO mice (n = 3–5 per genotype) during the first 6 months of life. (**I**) Appearance of sciatic nerves from control and Pbrm1 cKO pups (placed on a black background) at P21. Scale bar: 25 µm (**B**), 0.2 mm (**I**). Statistical significance was determined by unpaired two-tailed Student’s *t*-test (*P ≤ 0.05; ***P ≤ 0.001).

**Figure 3:**
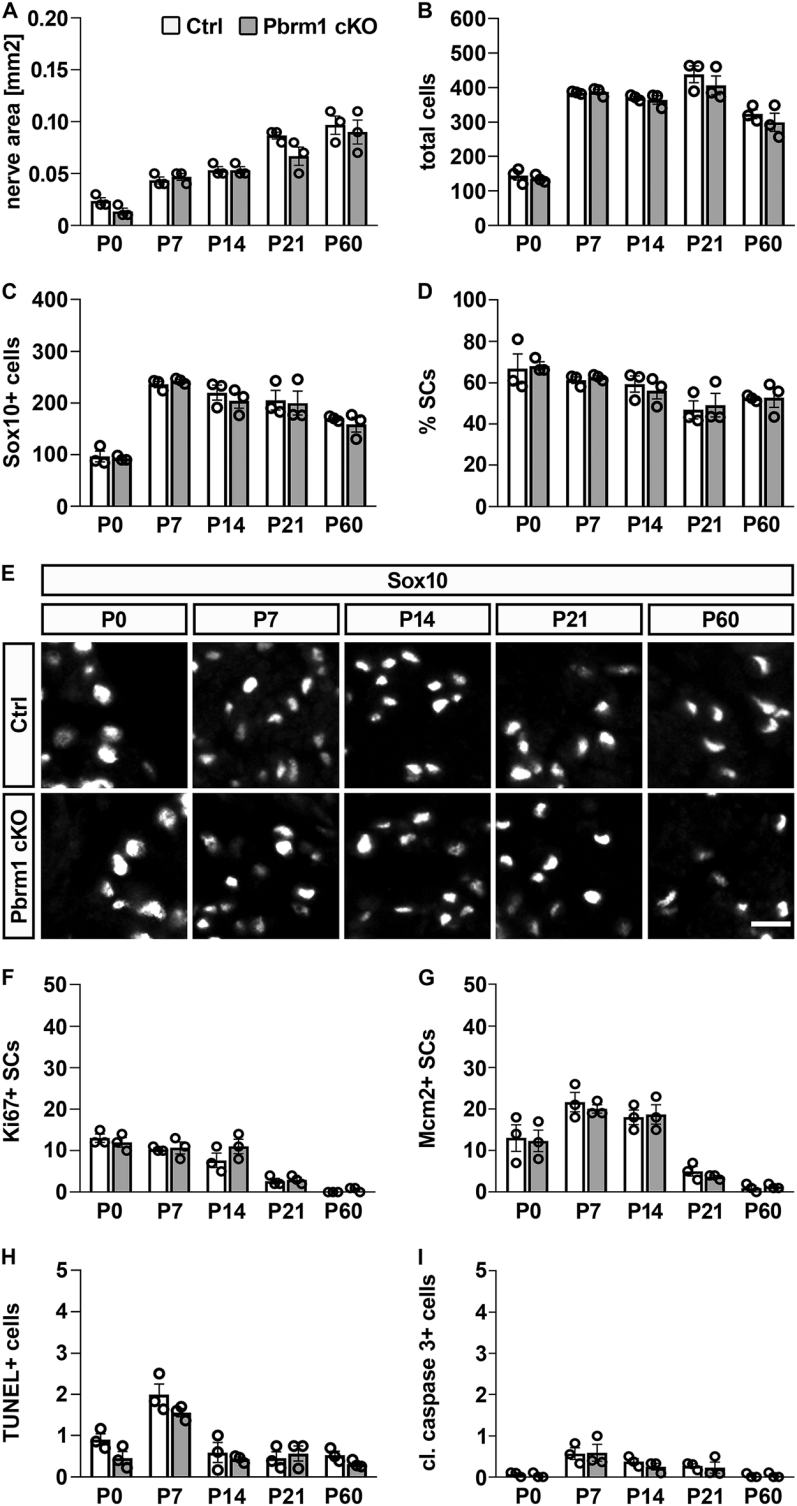
Schwann cells properties in sciatic nerves of Pbrm1 cKO mice. (**A**) Area of nerve cross-sections in square millimeters. (**B**–**D**) Absolute numbers of DAPI-positive nuclei (**B**) and Sox10-positive Schwann cells (**C**) per cross-section. Numbers were used to calculate the relative contribution of Schwann cells to the total nerve cell population (**D**) in sciatic nerves. (**E**) Immunohistochemical stainings of sciatic nerves of control and Pbrm1 cKO mice from P0 to P60 with antibodies directed against Sox10. Scale bar: 10 µm. (**F**, **G**) Absolute numbers of proliferating Schwann cells per cross-section by co-immunohistochemistry for Ki67 (**F**) and Mcm2 (**G**) with Sox10 antibodies. (**H**, **I**) Absolute number of dying cells per cross-section by TUNEL (**H**) and stainings for cleaved caspase 3 (**I**). All analyses were carried out during the first two postnatal months (P0–P60) on cross-sections of the tibial branch of the sciatic nerve at upper thigh level in control (Ctrl) and Pbrm1 cKO mice (shown as mean ± SEM, n = 3 per genotype). Statistical significance between genotypes was determined separately for each time point by unpaired two-tailed Student’s *t*-test. However, no significant difference was detected.

To confirm efficient deletion, we first convinced ourselves of the specificity of our anti-Pbrm1 antibodies. In western blots and immunocytochemical stainings, our antisera but not the preimmune sera specifically recognized the protein in Pbrm1-transfected HEK293 cells (Fig. 2A,B). Using these antisera, we determined a 97% deletion rate in sciatic nerve Schwann cells at P0 (Fig. 2C). Wildtype *Pbrm1* transcripts were also reduced to 46% in the sciatic nerve of Pbrm1 cKO mice at P7, in line with the fact that Schwann cells constitute a bit less than half of the nerve-associated cells (Figs. 2D, 3D). In contrast, transcript amounts for *Arid2*, *Brd7* and *Phf10* as other characteristic components of the PBAF complex were comparable in the sciatic nerve of control and Pbrm1 cKO mice at P7, P14 and P60 (Fig. 2E). Expression levels of the BAF complex subunits *Arid1b*, *Brd9* and *Dpf1* also remained unaltered arguing against a compensatory upregulation (Fig. 2F).

Pbrm1 cKO mice were born at the expected Mendelian ratio of 25% in breedings of *Pbrm1*^*fl/fl*^ with *Pbrm*^+*/fl*^* Dhh::Cre* mice and exhibited a postnatal growth that was indistinguishable from control littermates (Fig. 2G,H). Grip strength and the ability to hold onto an inverted cage lid were inconspicuous. There were no signs of hindlimb clasping. By overall appearance and opacity, sciatic nerves of Pbrm1 cKO mice and age-matched controls were indistinguishable as exemplified for P21 (Fig. 2I).

### Characterization of Schwann cell development in the absence of Pbrm1

To characterize Schwann cell development in more detail, we first determined sciatic nerve size and overall cell number from birth until two months of age. Judged by the area of the tibial branch in sciatic nerve sections at upper thigh levels and the number of DAPI-stained nuclei, no significant differences were evident between sciatic nerves of control and Pbrm1 cKO mice at any given time point (Fig. 3A,B).

Next we determined Schwann cell numbers during the postnatal period of active myelination in the sciatic nerve of Pbrm1 cKO mice. Using Sox10 as a marker for all Schwann cells, we counted similar absolute cell numbers from the day of birth until two months of age (Fig. 3C). By comparing Schwann cell to total cell numbers, it became evident that the overall contribution of Schwann cells to the nerve cell population was comparable between Pbrm1 cKO mice and controls (Fig. 3D). Distribution of Schwann cells throughout the nerve of Pbrm1 cKO mice was furthermore indistinguishable from controls as judged from the Sox10 stainings (Fig. 3E). In line with similar absolute and relative Schwann cell numbers, we also failed to detect substantial changes in the number of proliferating Schwann cells regardless of whether Ki67 or Mcm2 was used as marker (Fig. 3F,G). Cell death rates in sciatic nerves of Pbrm1 cKO mice were not significantly different from controls as determined by TUNEL and staining for cleaved caspase 3-positive cells (Fig. 3H,I). We conclude from these results that there are no substantial differences in Schwann cell numbers or their proliferation and survival upon loss of Pbrm1.

**Figure 4:**
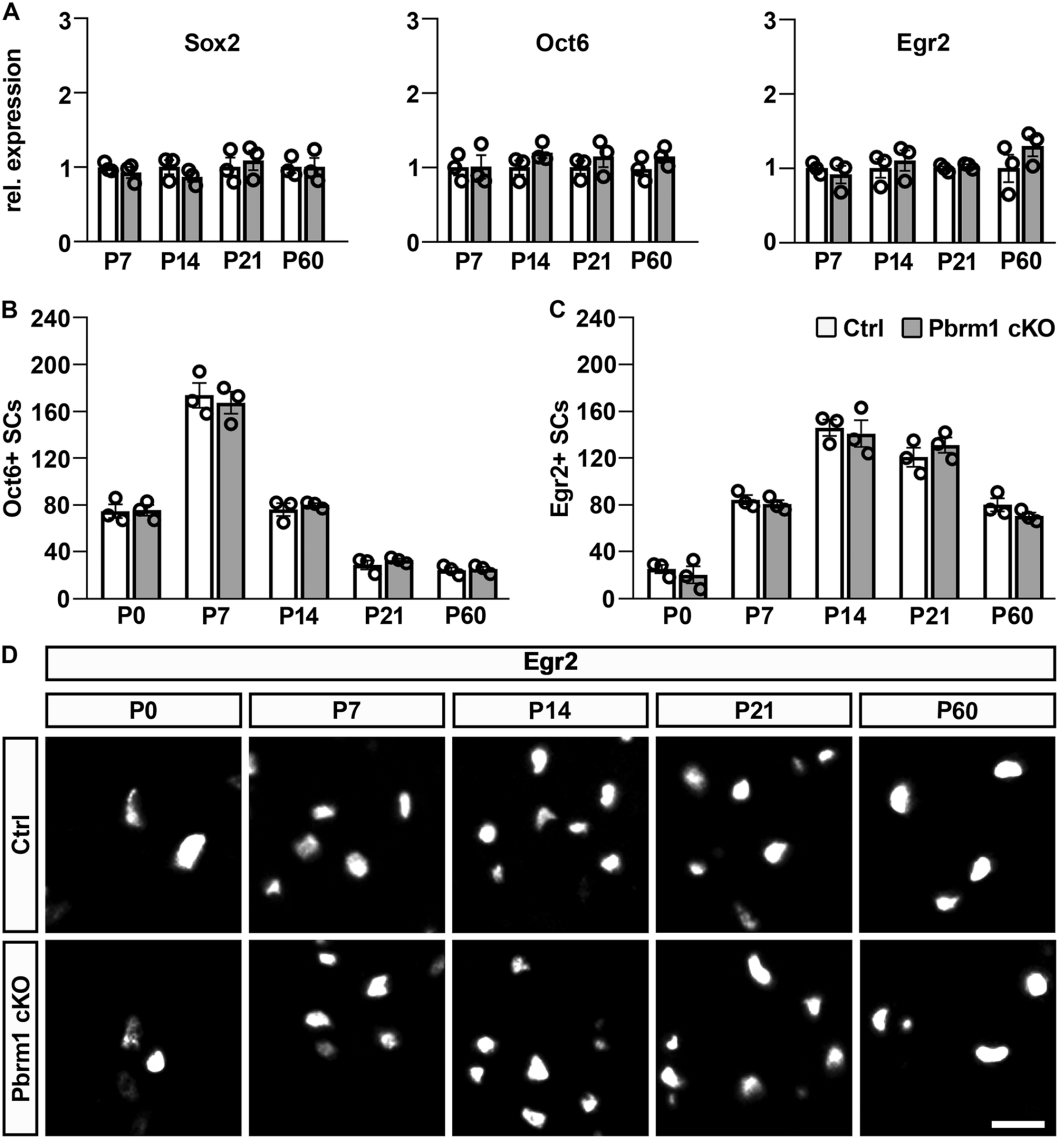
Schwann cell lineage progression in sciatic nerves of Pbrm1 cKO mice. (**A**) Quantitative RT-PCR of *Sox2*, *Oct6* and *Egr2* expression as stage-specific markers of Schwann cell development in sciatic nerves of control (Ctrl) and Pbrm1 cKO mice from P7 to P60 (shown as mean ± SEM, n = 3 per genotype). Average expression levels for each marker at each analyzed time point were set to 1. (**B**, **C**) Determination of Oct6-positive (**B**) pro-myelinating and Egr2-positive (**C**) myelinating Schwann cells per transverse sciatic nerve section in control and Pbrm1 cKO mice from P0 until P60 (shown as mean ± SEM, n = 3 per genotype). (**D**) Immunohistochemical staining of nerve sections of control and Pbrm1 cKO mice from P0 until P60 with antibodies directed against Egr2. Scale bar: 10 µm. Statistical significance between genotypes was determined separately for each time point and marker by unpaired two-tailed Student’s *t*-test. However, no significant difference was detected.

To characterize Schwann cell lineage progression, we next studied the occurrence of stage-specific markers in sciatic nerves of Pbrm1 cKO mice during the first two postnatal months on transcript levels. By quantitative RT-PCR, transcript levels for the immature Schwann cell marker *Sox2,* the pro-myelinating Schwann cell marker *Oct6* and the myelinating Schwann cell marker *Egr2* in sciatic nerves of Pbrm1 cko mice were comparable to controls at all time points analyzed during the first two months after birth (Fig. 4A).

Immunohistochemical studies confirmed a normal lineage progression. We were unable to detect significant differences in Oct6 protein expression between sciatic nerves of Pbrm1 cKO mice and age-matched controls (Fig. 4B). For this marker of the pro-myelinating stage, numbers were already elevated at P0 in both genotypes, increased even further at P7 and then steadily declined, reflecting the transition of pro-myelinating into myelinating Schwann cells. The rise of myelinating Schwann cells is also visible in the gradual increase of Egr2-positive cells from 25 ± 4 cells at P0 to 146 ± 7 cells at P14 in controls (Fig. 4C,D). From their peak at P14 and P21, numbers of Egr2-positive cells per nerve section slightly decreased at older ages concomitant with the substantial longitudinal extension of the sciatic nerve. Again, no substantial differences were detectable in Pbrm1 cKO mice (e.g. 20 ± 7 cells at P0 to 141 ± 12 cells at P14). We therefore conclude that lineage progression and stage specific marker gene expression are unaltered in Pbrm1-deficient Schwann cells.

### Characterization of myelin gene expression and myelination in the nerve of Pbrm1 cKO mice

**Figure 5:**
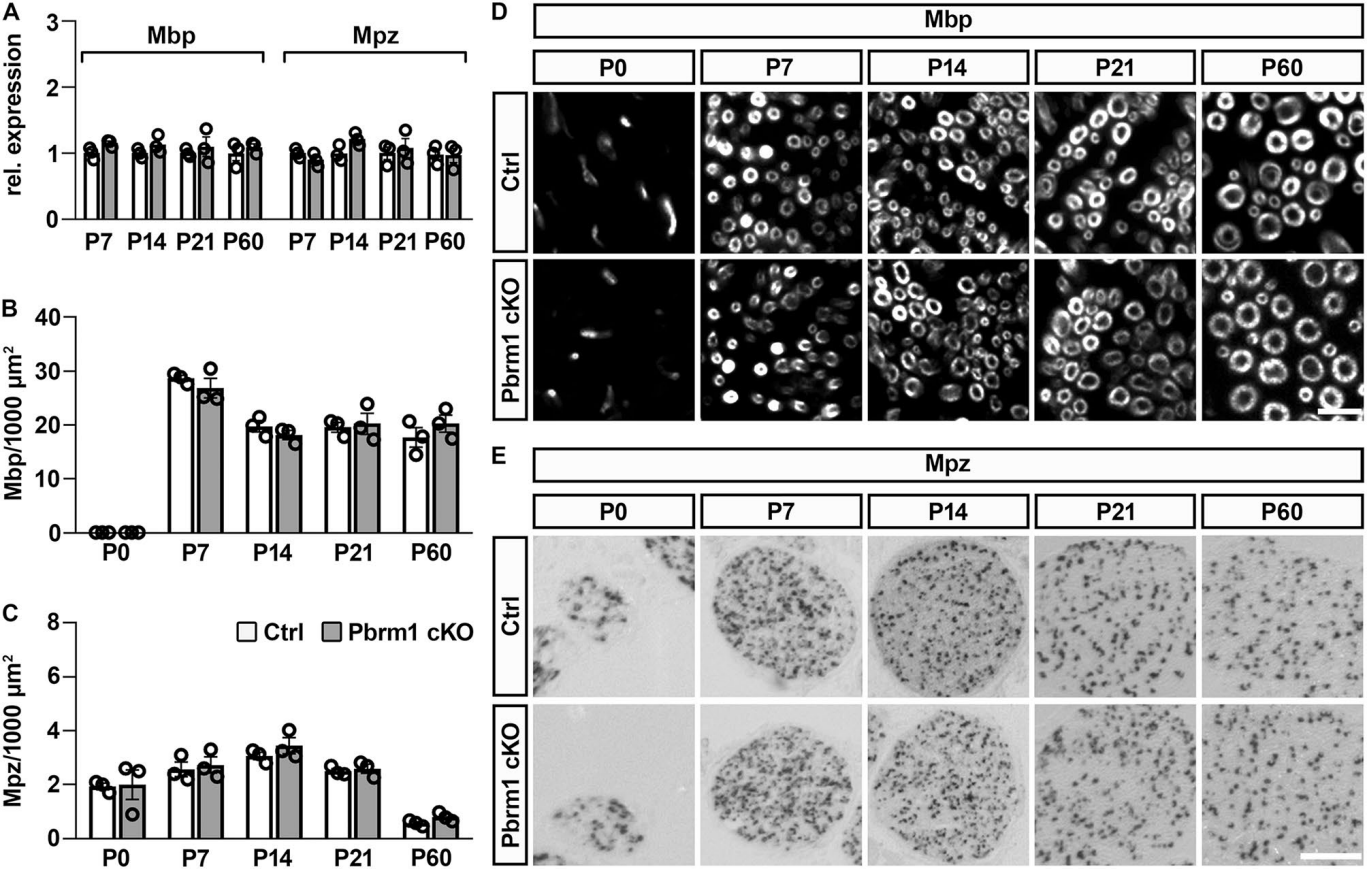
Myelin gene expression in sciatic nerves of Pbrm1 cKO mice. (**A**) Quantitative RT-PCR of *Mbp* and *Mpz* expression in sciatic nerves of control (Ctrl) and Pbrm1 cKO mice from P7 until P60 (shown as mean ± SEM, n = 3 per genotype). Average expression levels for each marker at each analyzed time point were set to 1. (**B**, **C**) Determination of Mbp-positive structures (**B**) and *Mpz*-expressing cells (**C**) per 1000 square micrometers in sciatic nerve sections of control and Pbrm1 cKO mice from P0 until P60 (shown as mean ± SEM, n = 3 per genotype). (**D**, **E**) Immunohistochemical staining for Mbp protein (**D**) and in situ hybridization with an *Mpz*-specific antisense riboprobe (**E**) of nerve sections from control and Pbrm1 cKO mice between P0 and P60. Scale bars: 10 µm (**D**), 500 µm (**E**). Statistical significance between genotypes was determined separately for each time point and marker by unpaired two-tailed Student’s *t*-test. However, no significant difference was detected.

Considering that Schwann cells develop normally and on schedule in Pbrm1 cKO mice, we next turned our attention to the myelination process. First, we analyzed myelin gene expression. By quantitative RT-PCR, overall transcript levels for *Mbp* and *Mpz* were comparable in sciatic nerves of control and Pbrm1 cKO mice (Fig. 5A). In addition to similar transcript levels, numbers of Mbp- and *Mpz*-expressing Schwann cells were highly alike in the sciatic nerve of both genotypes at all analyzed time points during the active phase of myelination as well as in two months-old young adults as determined by immunohistochemistry and in situ hybridization (Fig. 5B–E). We therefore conclude that myelin gene expression remains unaltered even after Pbrm1 loss in Schwann cells.

**Figure 6:**
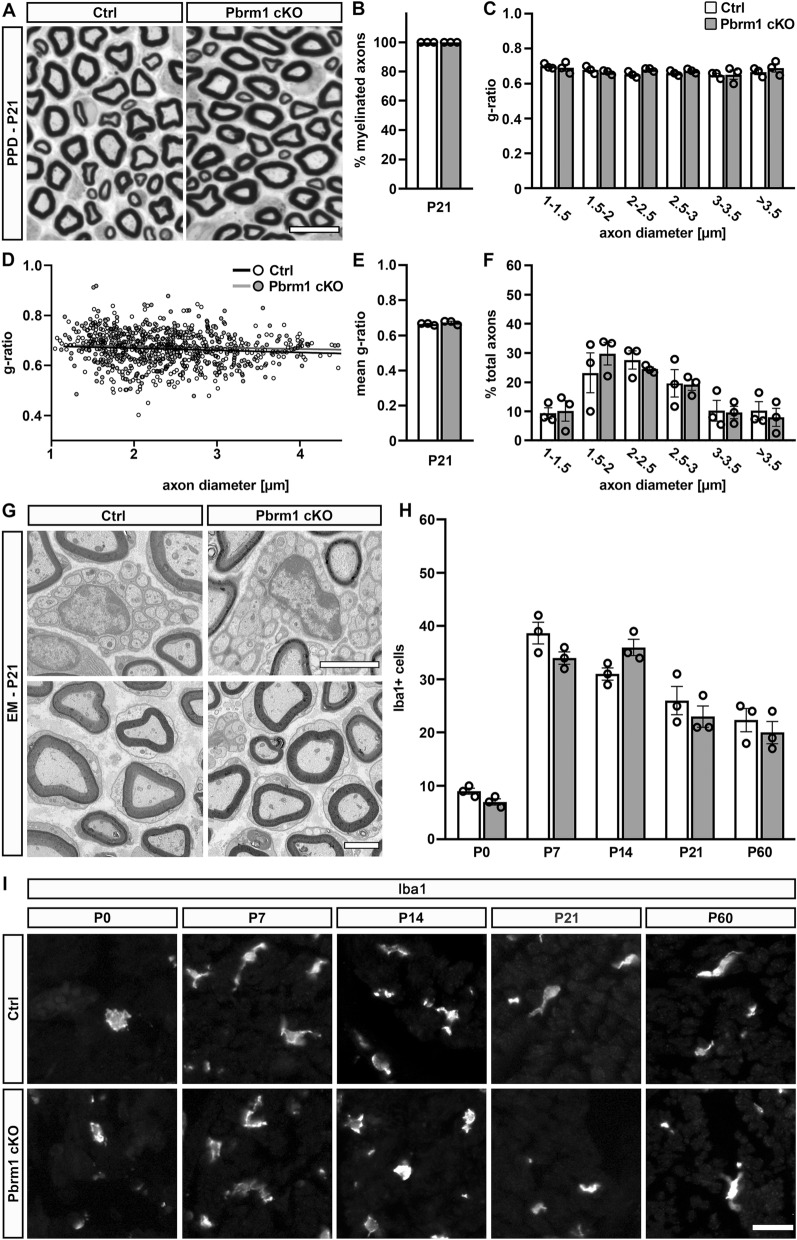
Myelination of sciatic nerves of Pbrm1 cKO mice at P21. (**A**) Overview of sciatic nerve tissue from control (Ctrl) and Pbrm1 cKO mice at P21 after PPD staining. (**B**) Quantification of the percentage of myelinated axons in sciatic nerves of control and Pbrm1 cKO mice at P21 (shown as mean ± SEM, n = 3 per genotype). (**C**–**E**) G-ratio determination for myelinated axons in sciatic nerves of control and Pbrm1 cKO mice after binning by axon diameter (**C**), as scatter blot for single fibers (**D**, based on n = 150 fibers per genotype) or as average (**E**, shown as mean ± SEM, n = 3 per genotype). (**F**) Percental size distribution of axons (larger than 1 µm) in sciatic nerves of control and Pbrm1 cKO mice at P21 after binning by diameter (n = 150 fibers per genotype). (**G**) High resolution electron microscopic pictures of sciatic nerve tissue from control and Pbrm1 cKO mice at P21 depicting a representative Remak bundle (upper panels) and myelinated large calibre axons (lower panels). (**H**) Determination of Iba1-positive macrophages per sciatic nerve cross-section in control and Pbrm1 cKO mice from P0 until P60 (mean ± SEM, n = 3 per genotype). (**I**) Immunohistochemical stainings of nerve sections of control and Pbrm1 cKO mice from P0 until P60 with antibodies directed against Iba1. Scale bar: 10 µm (**A**, **I**), 3 µm (**G**). Statistical significance between genotypes was determined separately for each time point, bin and marker by unpaired two-tailed Student’s *t*-test. However, no significant difference was detected.

For visualization of myelin structures, PPD stainings were performed on sciatic nerves at P21 (Fig. 6A). Light microscopic inspection of these stainings did not point to any obvious alterations in the degree of myelination and the appearance of myelin in sciatic nerves of Pbrm1 cKO mice. Quantifications confirmed that the percentage of myelinated axons among axons bigger than 1 µm was close to 100% in both genotypes at P21 (Fig. 6B). Neither binning of g-ratios by axon diameter nor a scatter blot of single fiber g-ratios revealed substantial deviations in Pbrm1 cKO mice from controls (Fig. 6C,D). The mean g-ratio was determined as 0.66 in controls and 0.67 in Pbrm1 cKO mice (Fig. 6E). Size distribution of myelinated axons was likewise comparable between both genotypes (Fig. 6F). Electron microscopic analysis confirmed a normal ultrastructure of myelin sheaths around large-caliber axons as well as intact Remak bundles (Fig. 6G). There were no signs of activated macrophages. This was corroborated by normal numbers of Iba1-positive macrophages in immunohistochemical stainings at all times of analysis (Fig. 6H,I). Thus, peripheral nerve myelination is largely normal even if Schwann cells lack Pbrm1.

## Discussion

In this study we have analyzed the consequences of *Dhh::Cre*-dependent Pbrm1 deletion in developing Schwann cells and have found no substantial alteration in the timing of lineage progression, survival and proliferation as well as in terminal differentiation and myelination. Similar results were also obtained at P0 and P7 when *Sox10::Cre* or *Cnp1::Cre* were employed instead of *Dhh::Cre* to delete Pbrm1 in Schwann cells. Pbrm1 has been shown to be an essential component of the vertebrate PBAF complex^9,12^. Normal development of Pbrm1-deficient Schwann cells therefore argues that the PBAF complex is not essentially required as chromatin remodeler during Schwann cell development and myelination.

The absence of major phenotypic alterations in Schwann cell development after Pbrm1 loss is in contrast to previous observations on the consequences of Brg1 deletion in Schwann cell development by the same Cre transgene^7^. Brg1 functions as the ATP-hydrolyzing and energy generating subunit in both the BAF and the PBAF complex^8^. Two scenarios can therefore explain the different phenotypes in the two mouse mutants. For one, it can be envisaged that Schwann cell development is only affected in the joint absence of the Brg1-containing BAF and PBAF complexes and that separate inactivation of either chromatin remodeler alone remains without major consequences. This would require each complex to largely compensate for the other. Alternatively, most of the effects previously observed after Brg1 deletion are due to its activity in the BAF complex rather than its activity in the PBAF complex.

Brg1 is recruited to Schwann cell-specific enhancers of the *Oct6* and *Egr2* genes as well as to enhancers of several myelin genes via Baf60a and its direct physical interaction with Sox10^7,17^. Baf60a is a subunit of the BAF complex but does not occur in the PBAF complex. Selective enhancer recruitment of the BAF complex may therefore explain its unique role in Schwann cell development.

In the central nervous system, oligodendrocytes represent the functional substitute of Schwann cells. In agreement with their divergent ontogenetic origin, Brg1 functions differently in oligodendrocytes and Schwann cells^7,18,19^ and it is by no means clear how oligodendroglial Brg1 functions partition between BAF and PBAF complexes. Future studies will have to clarify the role of Pbrm1 and the PBAF complex in oligodendrocytes and their development.

## Methods

### Generation and validation of Pbrm1 antibodies

Antibodies were raised in rabbit and guinea pig against a peptide that corresponded to amino acids 1288–1392 of mouse Pbrm1 by Pineda Antikörper-Service (Berlin, Germany). The peptide was produced in *E. coli* BL21 pLysS bacteria from a pET28a plasmid after IPTG induction and purified from whole bacterial extracts under denaturing conditions by an aminoterminally fused 6xHis-tag.

Antiserum from the final bleed was tested in western blot and immunochemical applications (Fig. 2A,B). For western blots, whole cell extracts from pCMV5-Pbrm1 transfected HEK293 cells were size-fractionated on a standard 10% polyacrylamide-sodiumdodecylsulfate-gel, transferred to a nitrocellulose membrane and incubated with antiserum at a 1:3000 dilution as previously described^7^. Antibody-specific signal detection was by horseradish peroxidase coupled to protein A and luminol reagent. For immunocytochemical stainings, HEK293 cells were seeded on cover slips, transfected with pCMV5-Pbrm1-GFP or pCMV5-GFP, paraformaldehyde-fixed after 48 h and analyzed for GFP and Pbrm1 expression by stepwise incubation with anti-GFP and anti-Pbrm1 antisera followed by fluorophore-coupled secondary antibodies (see section on immunohistochemical analysis) according to previously published protocols^7^. Antiserum specificity was further confirmed by immunohistochemistry on control and Pbrm1-deleted tissues (Fig. 2C).

### Husbandry and breeding of mice

Mice carrying floxed *Pbrm1* alleles (strain B6;129-Pbrm1tm1Zhwa/J)^15^ were obtained from the Jackson Laboratories (Bar Harbor, ME, USA) and were bred with mice expressing a *Dhh::Cre* transgene^16^. Genotyping was performed by PCR using 5′-GACATGGCTTCTCCCAAACT-3′and 5′-TGCAACTCTTTGTCCTTACACG-3′ as primers in 33 cycles of 30 s 94 °C, 40 s 60 °C and 60 s 72 °C for *Pbrm1*, and 5′-ATGCTGTTTCACTGGTTATG-3′ and 5′-ATTGCCCCTGTTTCACTATC-3′ as primers in 30 cycles of 30 s 94 °C, 30 s 54 °C and 30 s 72 °C for *Cre*. All mice were on a mixed C3H × C57Bl/6 J background and kept under standard housing conditions with continuous access to food and water in 12:12 h light–dark cycles. Timed matings were set up to generate litters at embryonic day (E) 12.5, 14.5, 16.5 and 18.5. Spinal nerves of embryos or sciatic nerves of mice from both sexes were processed for histological, immunohistochemical, and ultrastructural studies as well as RNA preparations as described^20^. Experiments were in accordance with animal welfare laws and ARRIVE guidelines and approved by the responsible local committees and government bodies (University, Veterinäramt Stadt Erlangen & Regierung von Unterfranken, TS-00/12 Biochemie II).

### Immunohistochemical analysis and in situ hybridization

Embryos and dissected sciatic nerve tissue underwent fixation in 4% paraformaldehyde, cryoprotection in 30% sucrose, embedding in Tissue Freezing Medium (Leica), and cryotome sectioning at 10 µm thickness before staining with the following primary antibodies: anti-Sox10 goat antiserum (1:3000 dilution, RRID:AB_2891326)^21^, anti-Sox10 guinea pig antiserum (1:1000 dilution, RRID:AB_2721917)^22^, anti-Egr2 guinea pig antiserum (1:1000 dilution, RRID:AB_2891327)^6^, anti-Pbrm1 guinea pig antiserum (1:5000 dilution), anti-Pbrm1 rabbit antiserum (1:3000 dilution), anti-Oct6 rabbit antiserum (1:2000 dilution, RRID:AB_2891333)^23^, anti-CD3 rabbit antiserum (Abcam, #ab5690, Lot#665620, 1:500 dilution), anti-Iba1 rabbit antiserum (Wako, #019-19741, Lot#SAE6921, 1:250 dilution), anti-Desmin rabbit antiserum (Abcam, #ab15200, 1:1000 dilution), anti-fibronectin rabbit antiserum (Abcam, #ab2413, Lot#GR3235936-2, 1:100 dilution), anti-Gfp chicken antiserum (Aves Labs, #GFP-1020, Lot#GFP879484, 1:2000), anti-cleaved caspase 3 rabbit antiserum (Cell Signaling Technology, #9661, Lot#0043, 1:200 dilution), anti-Ki67 rabbit antiserum (Thermo Fisher Scientific, #RM-9106, Lot#9106S906D, 1:500 dilution), anti-Mcm2 rabbit antiserum (Cell Signaling Technology, #4007, Lot#3, 1:200 dilution), anti-MBP rat monoclonal antibody (Bio-Rad, #MCA409S, Lot #210610, 1:500 dilution, RRID:AB_325004), and anti-α smooth muscle actin mouse monoclonal (Sigma, #A5228, 1:200 dilution). Secondary antibodies coupled to Cy3, Cy5 or Alexa488 dyes (Dianova Cat# 705-175-14, 705-165-147, 706-165-148, 706-175-148, 706-545-148, 711-165-152, Invitrogen Cat# A11055 and A21206, Jackson ImmunoResearch Labs Cat# 711-175-152, at 1:200 to 1:500 dilution) were used for fluorescence labeling. Nuclei were counterstained with 4′,6-diamidino-2-phenylindole (DAPI, Sigma Cat# D9542). TUNEL was performed by using the ApopTag Red In Situ Apoptosis Detection Kit according to the manufacturer’s protocol (Chemicon). A digoxigenin-labeled antisense riboprobe specific for *Mpz* (positions 1–1864 acc. to accession number NM_001314068) was used for in situ hybridization^6^. Documentation of fluorescent signals was with a DMI 6000B inverted microscope (Leica) coupled to a DFC 360FX camera (Leica). For quantifications, 3 sciatic nerves were analyzed. For each nerve, the mean was determined by calculating the area of three cross-sections from the tibial branch at upper thigh levels and counting all fluorescent signals obtained in particular stainings and the DAPI counterstain. Error bars represent standard error of the mean (SEM).

### Histology and electron microscopy

Dissected sciatic nerves were successively incubated in cacodylate-buffered fixative containing 2.5% paraformaldehyde and 2.5% glutaraldehyde and in cacodylate-buffered 1% osmium ferrocyanide, dehydrated and embedded in Epon resin. Sectioning was at 1 µm for para-phenylene-diamine (PPD) stainings (Carl Roth, Karlsruhe, Germany) or at 50 nm for staining with uranyl acetate and lead citrate. PPD stainings were analyzed by a Leica DMR microscope and used to determine the number of myelinated axons. Stainings with uranyl acetate and lead citrate were studied under a Zeiss Libra electron microscope (Carl Zeiss, Inc.) for analysis of nerve ultrastructure. Axonal perimeter as well as the perimeter of the surrounding myelin sheath were measured using Fiji^24^. Perimeters were subsequently used to calculate the respective diameters and determine the g-ratios (defined as the ratio of the inner axonal diameter and the outer, myelinated axonal diameter.

### RNA preparation, reverse transcription and quantitative PCR

After addition of 1 ml Trizol reagent sciatic nerves were dissociated using gentleMACS Dissociator (Miltenyi Biotec) and RNA was prepared according to the manufacturer’s protocol (Invitrogen Cat# 15596018). RNA samples were reverse transcribed and underwent quantitative PCR on a Bio-Rad CFX96 Real-Time PCR Detection System using the PowerUp SYBR Green Master Mix (Thermo Fisher Scientific, Darmstadt, Germany). Expression levels were determined according to the ΔΔCt method. Primer pairs have been described^6,21^ except for *Pbrm1* (5′-CAATGAGCCTGGGTCTCAAGT-3′ and 5′-CGGTAATACTTGCTAGTGGGGT-3′), Arid1b (5′-CTTCCTAGAGGACGGGGTGA-3′ and 5′-TCGTGCAAAAGGAACTCCGA-3′), Arid2 (5′-AACCCTCGCAGTCACGAAAA-3′ and 5′-TGCCAACAGAGCACTAGGC-3′), Brd7 (5′-ACAGCAAGTGACTCCAGGTG-3′ and 5′-CTAGTTCAGCTCGCGTCAGG-3′), Brd9 (5′-CAGGCTCCTGCAGGACTTAC-3′ and 5′-ATTCATAAGGGTCGTGGGCG-3′), Dpf1 (5′-GTCTCCGGCACTTGAAGGAA-3′ and 5′-CTTCACCCCATCCCTTCCAC-3′) and Phf10 (5′-GACAGCCCCACCATGAAGAA-3′ and 5′-GCTGTTTTTCCCCCTTCTGC-3′) using 40 cycles of 10 s 95 °C, 30 s 60 °C, 45 s 60 °C for amplification*.* Levels of all transcripts were normalized to *Rplp0* and *Gapdh* levels. Normalized levels for a particular transcript at a given time point in proliferating Schwann cell cultures or control sciatic nerve samples were arbitrarily set to 1 and the corresponding values for differentiating Schwann cell cultures or samples from Pbrm1 cKO mice were set in relation to it. Absolute read numbers from deposited RNA-Seq studies (GSE101153) were additionally used as measure of expression levels.

### Statistical analysis, preparation of graphs and figures

Whenever possible, experiments and analyses were carried out in a blinded fashion. No sample calculation was performed. Sample size was set to n = 3–5 as common for such studies, if not otherwise stated. Results from independent specimens were treated as biological replicates. To determine whether differences in cell numbers or amounts were statistically significant (*P ≤ 0.05; **P ≤ 0.01, ***P ≤ 0.001), an unpaired two-tailed Student’s t test was performed, in which matching data sets (i.e. from sciatic nerve of control and Pbrm1 cKO mice at a particular age or proliferating and differentiating Schwann cells from the same preparation) were compared. For statistical analyses and creation of all graphs in Figs. 1, 2, 3, 4, 5 and 6 GraphPad Prism version 8 (GraphPad Software, La Jolla, CA, USA; www.graphpad.com/scientific-software/prism) was used. All other panels were prepared using Adobe Photoshop CS2 (Adobe Inc., San Jose, CA, USA; www.adobe.com/de/products/photoshop).

## Data Availability

All data generated and analyzed during this study are included in this article.
